# Silicon nanopore membrane (SNM) for islet encapsulation and immunoisolation under convective transport

**DOI:** 10.1038/srep23679

**Published:** 2016-03-24

**Authors:** Shang Song, Gaetano Faleo, Raymond Yeung, Rishi Kant, Andrew M Posselt, Tejal A Desai, Qizhi Tang, Shuvo Roy

**Affiliations:** 1 Department of Bioengineering and Therapeutic Sciences, University of California–San Francisco, San Francisco, CA, 94158, United States; 2 Department of Surgery, University of California–San Francisco, San Francisco, CA, 94143, United States

## Abstract

Problems associated with islet transplantation for Type 1 Diabetes (T1D) such as shortage of donor cells, use of immunosuppressive drugs remain as major challenges. Immune isolation using encapsulation may circumvent the use of immunosuppressants and prolong the longevity of transplanted islets. The encapsulating membrane must block the passage of host’s immune components while providing sufficient exchange of glucose, insulin and other small molecules. We report the development and characterization of a new generation of semipermeable ultrafiltration membrane, the silicon nanopore membrane (SNM), designed with approximately 7 nm-wide slit-pores to provide middle molecule selectivity by limiting passage of pro-inflammatory cytokines. Moreover, the use of convective transport with a pressure differential across the SNM overcomes the mass transfer limitations associated with diffusion through nanometer-scale pores. The SNM exhibited a hydraulic permeability of 130 ml/hr/m^2^/mmHg, which is more than 3 fold greater than existing polymer membranes. Analysis of sieving coefficients revealed 80% reduction in cytokines passage through SNM under convective transport. SNM protected encapsulated islets from infiltrating cytokines and retained islet viability over 6 hours and remained responsive to changes in glucose levels unlike non-encapsulated controls. Together, these data demonstrate the novel membrane exhibiting unprecedented hydraulic permeability and immune-protection for islet transplantation therapy.

Type 1 diabetes (T1D) results from autoimmune destruction of the insulin-producing β-cells within the pancreatic islets of Langerhans. Islet transplantation by direct infusion of cadaveric islets into the portal vein of the recipient’s liver offers a non-invasive cure for patients with T1D mellitus^1^. However, donor availability, poor engraftment, and side effects from global immunosuppression remain as obstacles for wider application of this approach^2^,^3^,^4^. Moreover, up to 60% of the infused islets become non-viable within a few days after surgical delivery^5^ and the long-term insulin independence is frequently lost by 5 years of transplantation^6^. The activation of innate and the adaptive immune responses are among the main causes of islet graft failure^7^,^8^.

The idea of encapsulating islets using selective semi-permeable membranes to protect islets from the host’s immune system has generated tremendous interest^9^. The immunoisolating membranes would prevent the passage of the host’s immune factors, while allowing the exchange of glucose, insulin, nutrients and small molecules to sustain the function and viability of the graft. Although membranes with pores smaller than 1 μm can easily block immune cells (~10 μm), the blockage of molecules such as antibodies and cytokines proves to be a significant challenge. Previous studies showed that large antibody (IgM) and complement (C1q) were hindered using membranes with a maximum pore diameter of 30 nm^10^. For cytokines, the membranes must selectively discriminate between molecules on the scale of few nanometers, as shown by the molecular weights and Stokes diameters in Tumor Necrosis Factor-alpha (TNF-α) (17,300 Da; 3.80 nm)^11^,^12^, and Interferon-gamma (IFN-γ) (15,600 Da; 3.67 nm)^12^,^13^, and Interleukin-1 beta (IL-1β) (17,500 Da; 3.81 nm)^14^,^15^ compared to glucose (180 Da; 0.82 nm)^12^,^16^ and insulin (5,800 Da; 2.64 nm)^12^,^17^. These cytokines are known to be synergistically cytotoxic to islets through a cascade of inflammatory events such as production of nitric oxide (NO) and chemokines, and trigger of endoplasmic reticulum stress^18^,^19^. Conventional polymeric membranes face enormous challenge for size-dependent separation of these cytokines as polymeric membranes frequently exhibit pore sizes with relatively broad distributions (30%)^20^.

Our lab has developed a new generation of encapsulating membranes for immunoisolation of transplanted islets based on microelectromechanical systems (MEMS) technology initially pioneered by Ferrari and colleagues^21^,^22^ to create more uniform pore sizes at nanometer scale. These semipermeable filtration membranes, termed silicon nanopore membranes (SNM), can be engineered with precise pore sizes down to 5 nm (Fig. 1)^23^ and a monodisperse pore size distribution (~1%) for superior selectivity^20^,^23^,^24^,^25^. The ability to engineer precise pore dimensions in a uniform manner enables SNM to discriminate larger immune components from smaller molecules that will pass into the encapsulated cells. When pore dimensions are of the same order as those of a solute molecule^26^, the slow diffusion significantly hinders transport of nutrients and oxygen. In contrast, convective transport is attractive as it offers a more efficient mass transfer where solutes actively move along with solvent flux due to applied pressure gradient. Our overall objective is an implantable bioartificial pancreas where transplanted islets are encapsulated between two SNM sheets in a device that will be mounted similarly to an artero-venous (AV) graft (Supplementary Fig. S1). The concept involves using the pressure difference between the artery and vein to generate ultrafiltrate and drive transport of glucose, insulin, and other small molecules through the SNM to support function of encased islets while preventing passage of immune components.

In this study, we focused on SNM design and fabrication, followed by characterization of its immunobarrier properties under cytokine challenge with convective transport, and assessment of SNM-encapsulated islet viability and glucose-insulin response. Specifically, hydraulic permeability measurement and solute selectivity for SNM were determined. Mouse islets were encapsulated between SNM in a closed mock-loop fluid circuit (Supplementary Fig. S2) under simulated physiological pressure difference in the presence of a cocktail of pro-inflammatory cytokines including TNF-α, IL-1 β, and IFN-γ. Islet viability and glucose stimulated insulin production were evaluated to demonstrate the potential of SNM as an encapsulation material for islet immunoisolation under convective transport.

## Results and Discussion

MEMS fabrication technologies offers unprecedented potential in reproducibility and precision to engineer controlled pore dimensions that can selectively block the passage of immune components while allowing transport of small molecules (e.g. glucose and insulin) to sustain the viability of the encased cells. In the present study, we characterized the permeability and selectivity of the SNM to prevent cytokine infiltration and assessed the functional performance of SNM-encapsulated mouse islets in a mock-loop device under convective transport.

### SNM design and fabrication

Previously, Desai *et al*. reported silicon-based micromachined nanochannels that consisted of L-shaped pore paths with nanochannels running parallel to the membrane surface^27^,^28^,^29^,^30^,^31^. Although the design was effective in preventing diffusion of larger immunogenic molecules, the L-shaped path drastically reduced diffusion of smaller molecules of interest because of the long, indirect flow path and the less optimal pore density stemming from the large area per pore. The L-shaped pore design was also utilized in the islet-encapsulating Nanogland device, in which laterally positioned 3.6 and 5.7 nm nanochannels produced a reduction in glucose diffusivity by 40% and 25% respectively compared to the diffusivity in bulk medium^32^.

**Figure 1:**
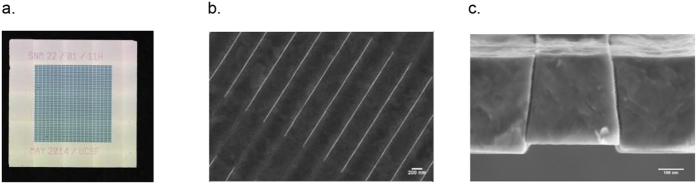
Silicon nanoporous membranes (SNM). (**a**) an optical image of the SNM chip. (**b**) An SEM image of the surface of the membrane which illustrates nanopores with 2 μm in length. (**c**) An SEM image of the cross-section of the membrane which illustrates one nanopore with 7 nm in width and 300 nm in depth.

**Figure 2:**
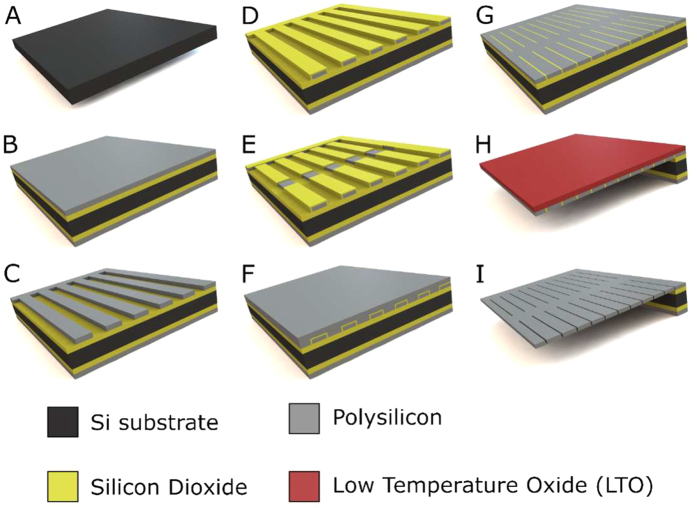
Schematic for fabrication of silicon nanopore membranes. (**A**) Piranha clean of double side polished Si wafer. (**B**) Thermal oxidation growth of SiO_2_ and low pressure chemical vapor deposition (LPCVD) of polysilicon. (**C**) Dry-etch patterning of polysilicon. (**D**) Thermal oxidation growth of SiO_2_ for use as sacrificial layer defining nanopores. (**E**) Patterning of anchor layer by wet etch. (**F**) LPCVD of polysilicon. (**G**) Blanket-etch of polysilicon until exposure of vertical SiO_2_ nanopores. (**H**) Deposition of low temperature oxide (LTO) for membrane protection and backside etch of membrane with deep reactive ion etching. (**I**) Dry etch removal of LTO and wet etch release of SiO_2_.

We have engineered a new generation of semipermeable membranes, SNM, with slit-pore designs initially investigated by Desai *et al*.^21^,^22^. The SNM exhibit a pore size distribution of ~1%^13^,^16^,^17^,^18^ and a consistent pore size control in the range of 5-15 nm^23^ (Fig. 1). The slit pore microarchitecture of SNM was achieved by dry oxidation of polysilicon for the growth of silicon dioxide (SiO_2_) (Fig. 2D) and through backside patterning with deep ion-reactive etching (DRIE) which resulted in vertical sidewalls in each membrane window (Fig. 2H). This process allows for fabrication of membranes with greater number of exposed nanopores per area compared to those with v-shaped sidewalls achieved by anisotropic KOH etching used by Desai *et al*.^27^. SNM were produced with an active membrane area (6 × 6 mm) consisting of ~10^6^ rectangular slit pores with ~7 nm in width, 300 nm in depth, and 2 μm in length (Fig. 1). The travel path could be further optimized by lowering the thickness of the membrane which can easily be controlled by the thin film low-pressure chemical vapor deposition (LPCVD) (Fig. 2B) or dry etch process (Fig. 2G). The utilization of a sacrificial layer to define the nanopores resulted in a membrane with a straight slit-pore path that presents a shorter distance for molecules to travel compared to the previous “L” pore design. The pore geometry could further influence the trade-off between selectivity and permeability of the membranes. The permeability – selectivity analysis for ultrafiltration demonstrated that membranes with slit-shaped pores showed higher performance and greater selectivity at a given value of permeability, than membranes with cylindrical pores for pore size below 100 nm^24^. To circumvent the slow concentration-dependent diffusion occurred in size-restricted nanoporous membranes, the concept of using convection-dominated transport is more advantageous in terms of creating faster solvent movement under transmembrane pressure gradient^33^, which efficiently drags small molecules such as glucose and insulin across membranes to the encapsulated cells.

### SNM permeability and selectivity characterization

**Figure 3:**
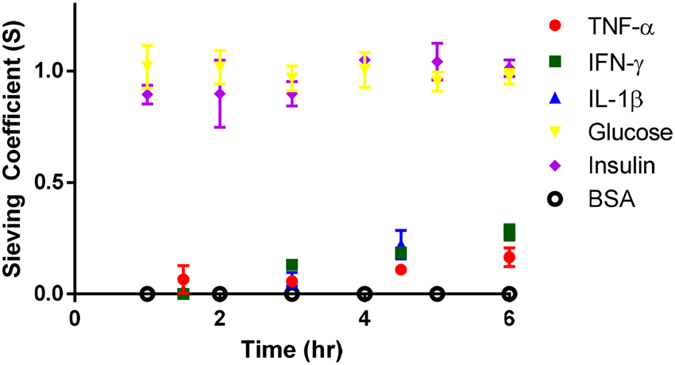
Transport of various molecules through slit-pore of SNM under a pressure difference of ~2 psi. Sieving coefficients (**S**) were expressed as the ratio of the concentration of the filtrate over the concentration of the feed (means ± SE). BSA was used as a negative control. Results showed that the sieving coefficients of TNF-α, IFN-γ, and IL-1β were 0.16, 0.27, and 0.27 after 6 hours, respectively. The sieving coefficients of glucose and insulin quickly reached 1. These data indicated that small molecules such as glucose and insulin completely passed the SNM whereas the entry of cytokines was greatly hindered under convective transport.

Permeability and selectivity of the SNM were characterized with the hydraulic permeability testing setup (Supplementary Fig. S3), which uses liquid flow through planar nanoporous membranes under tangential-flow filtration operation^34^. We demonstrated that SNM with pore sizes of 7 nm generated a hydraulic permeability of 130 ml/hr/m^2^/mmHg, which is much greater compared with conventional polymer membranes (~40 ml/hr/m2/mmHg) used in previous bioartificial pancreas devices^35^. To further demonstrate the feasibility of SNM for immunoisolation, we then characterized the membrane selectivity against transport of cytokines and small molecules using the pressure-driven ultrafiltration system (Supplementary Fig. S4). Solute transport was evaluated at ~2 psi driving pressure to mimic the typical physiological pressure difference between artery and vein^36^, which results in an ultrafiltration rate of ~4 ul/min. The membrane Peclet number (Pe) for the pressure-driven ultrafiltration system was significantly greater than 1, suggesting that convective transport dominates. The observed sieving coefficients (calculated using Eq. 1) should reflect the rejection characteristics of the membrane^37^. After 6 hours, the sieving coefficients of TNF-α, IFN-γ, and IL-1β were 0.16, 0.27, and 0.27, respectively (Fig. 3). In contrast, the sieving coefficients of glucose and insulin quickly reached 1 (Fig. 3). These data collectively demonstrate that SNM provide about 80% rejection of cytokine passage, while allowing complete transport of small molecules. Because concentration polarization and transmembrane diffusion were negligible in this experimental system, the observed sieving coefficient should be equal to the product of the solution partition coefficient (Ф) and the convective hindrance factor (K_c_). Previously, Dechadilok and Deen derived an analytic expression for the product of ΦK_c_ which describes a rigid sphere passing in a slit-shaped pore: 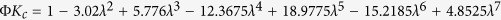 (Eq. 2)^38^, where λ is the relative solute size indicating the ratio between the diameter of the molecule and the width of slit-pore channel. Based on the observed sieving coefficients of cytokines (Fig. 3), we can calculate the corresponding relative solute sizes λ from Deen’s model (Eq. 2) for TNF-α, IFN-γ, and IL-1β as 0.83, 0.74, and 0.74, respectively. The experimental relative solute sizes of these cytokines are larger than the theoretic values, as indicated by Stokes-Einstein’s radius^12^ (Supplementary Fig. S5). This difference in relative solute sizes between the experimental and theoretical values could be explained by the fact that cytokines are not strictly spherical: TNF-α is a packed cubic shape consisting of trimers formed with β-sheet structure^39^, IFN-γ is a globular dimer with flattened elliptical shaped subunits^40^, and IL-1β has β-strands wrapped around in a tetrahedron-like fashion^41^. Furthermore, the electrostatic interactions associated with diffuse electrical double layer (EDL) around charged proteins could also increase the overall molecule size^42^,^43^, thereby overestimating the experiment relative solute sizes.

In summary, the SNM enables higher levels of ultrafiltrate production and demonstrate selective rejection against middle molecules like cytokines. Therefore, by encapsulating islets in SNM, we postulate that the increased convective mass transport of nutrients and glucose can support islet viability and insulin production, while the selective rejection of immune components enables exceptional immunoisolation.

### Assessment of SNM-encapsulated islets cultured under mock-loop circuit

The feasibility of developing an implantable SNM-encapsulated bioartificial pancreas device using convective transport was demonstrated using a mock-loop setup. The middle cell chamber is sandwiched between two membranes to closely mimic the *in vivo* conditions where SNM-encapsulated islets will be mounted as an arterio-venous (AV) graft (Supplementary Fig. S1). The pressure difference between the artery and vein will generate the ultrafiltrate and drive transport of water, salts, glucose, insulin, and other small molecules through the SNM, while passage of immune components such as cytokines will be blocked.

**Figure 4:**
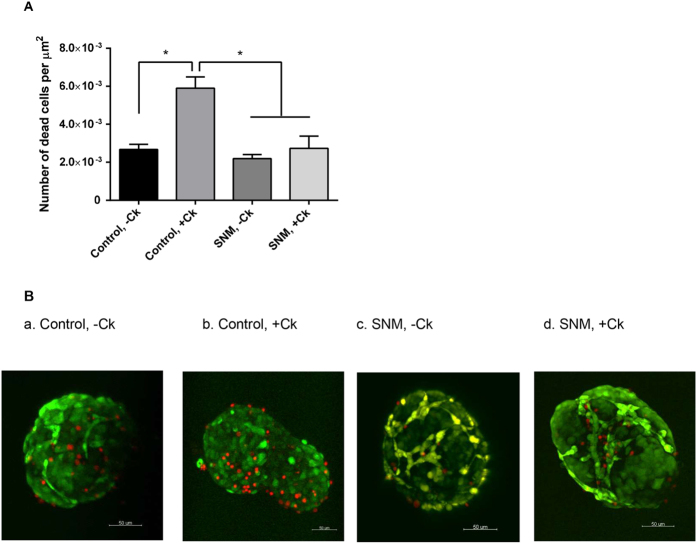
*In vitro* viability of mouse islets under cytokine exposure. (**A**) Viability of SNM-encapsulated mouse islets was measured following the 6 hr experiment in which islets were subjected to culture solution circulating the mock-loop circuit at 5 ml/min with a pressure difference of 2 psi. (**B**) Viable (green) and dead (red) cells were stained for control static culture (**a,b**) and SNM-encapsulated mouse islets (**c,d**). Experiments with cytokine exposure (indicated by +Ck) consisted of media containing TNF-α, IFN-γ, and IL-1β. The viability of islets was calculated based on the ratio of dead cells (in red) over the islet area. Viabilities of islets in static cultures were evaluated as control for comparison. SNM protected encapsulated mouse islets from pro-inflammatory cytokines (SNM, +Ck), which showed similar viability to SNM-encapsulated mouse islets without cytokine exposure (SNM,−Ck) and control static culture without cytokine exposure (Control,−Ck). Control static culture with cytokine exposure (Control, +Ck) showed significantly more cell death compared with other groups. (n > 3, *p < 0.05).

After passing the cytokine-contained media from the reservoir through the mock-loop circuit for 6 hr under applied physiological pressure ~2psi^36^, samples that were collected from the top, middle, and bottom chambers of the flow cell device were compared against the reservoir concentration. The level of cytokines TNF-α, IFN-γ, and IL-1β were significantly reduced to 30%, 35%, and 34% in the middle chamber, whereas small molecules insulin and glucose passed completely (~100%) through both membranes (Supplementary Fig. S6). To further examine the SNM-encapsulated islets under convective transport in the proposed mock-loop circuit, mouse islets were loaded into the middle chamber with or without cytokine circulation for 6 hr. The static culture incubated with cytokines showed a more than 2.2-fold increase in cell death compared to the static culture without cytokines, mock-loop device without cytokines, and mock-loop flow cell device with cytokines (Fig. 4). Moreover, no significant change in islet viability was observed among the static culture without cytokines, mock-loop device without cytokines, and mock-loop flow cell device with cytokines (Fig. 4). This demonstrated the effectiveness of SNM to protect islets from pro-inflammatory cytokine attack maintaining islet viability.

**Figure 5:**
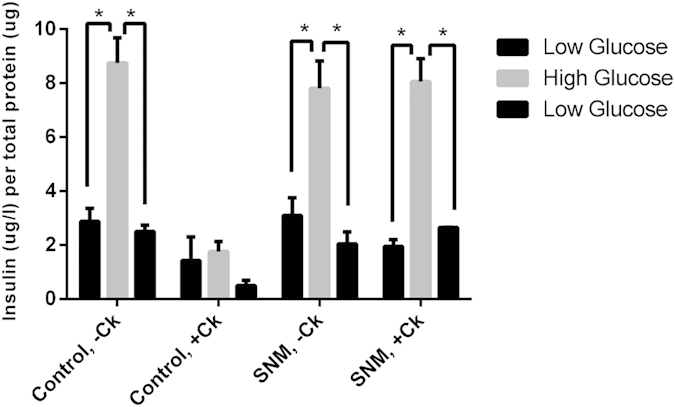
Glucose-stimulated insulin release of mouse islets in the SNM-encapsulation chamber and in static culture. Islets were subjected to media containing low-glucose, high-glucose, and low-glucose for 15 min each. Experiments with cytokine exposure (indicated by +Ck) consisted of culture solution containing TNF-α, IFN-γ, and IL-1β. The static culture without cytokines (Control,−Ck), mock-loop device without cytokines (SNM,−Ck), and mock-loop flow cell device exposed with cytokines (SNM, +Ck) had a 3.0-fold, 2.6-fold, and 4.1-fold increase in the amount of insulin secreted during high glucose challenge over those secreted during low glucose phase, respectively. However, the control static culture with cytokine exposure (Control, +Ck) secreted limited amount of insulin upon high glucose challenge due to the dead cells damaged by cytokine infiltration. (n > 3, *p < 0.05).

Additionally, the static culture without cytokines, mock-loop device without cytokines, and mock-loop flow cell device with cytokines demonstrated a 3.0-fold, 2.6-fold, and 4.1-fold changes, respectively, in the amount of insulin secreted during high glucose challenge compared with those secreted during low glucose challenge, respectively (Fig. 5). However, the static culture incubated with cytokines exhibited little variation in insulin secretion upon changes in glucose level (Fig. 5) due to loss in islet viability (Fig. 4). The glucose challenge demonstrated that the SNM-encapsulated mouse islets responded properly to changes in glucose level, whereas cytokine-infiltrating mouse islets lost their insulin-secreting ability to sense glucose stimuli. These data confirmed the usefulness of SNM to provide desired immunoisolation to support the viability and functional performance of the encapsulated islets.

In this study, we have developed and characterized an improved silicon nanopore membrane, SNM, for the encapsulation of pancreatic islets under convective flow. The SNM structure was specifically designed to obtain a well-defined slit pore in the nanometer range with a remarkably high hydraulic permeability. Furthermore, we have showed for the first time that SNM achieved high molecule selectivity against middle molecules such as cytokines under convective transport and provided adequate immune-protection to the encapsulated islets while generating sufficient filtrate to support viability and functionality of the encapsulated islets. Successful islet encapsulation with SNM could potentially reduce the immunosuppressive drugs and their side effects resulted from current therapies and lead to the possibility of encasing xenogeneic and stem-cell derived cell sources to overcome donor shortage for T1D treatment in the future. Further work is needed to optimize the SNM, including configuration of the slit-pores and extra reduction of membrane thickness, and study SNM-encapsulated graft performance and its immune-barrier function *ex vivo*.

## Methods

### Experimental overview

SNM were fabricated to produce an active membrane area (6 × 6 mm) consisting of ~10^6^ rectangular slit pores with ~7 nm in width, 300 nm in depth, and 2 μm in length (Fig. 1). The surface of SNM was subsequently modified with polyethylene glycol (PEG) to minimize protein fouling^44^. All SNM membranes in this study were tested with an average pore size of ~7 nm. We first analyzed the transport of small solutes including cytokines across a single SNM using a pressure-driven filtration assembly (Supplementary Fig. S4). To mimic the proposed bioartificial pancreas device with convective ultrafiltration under physiological pressure, we constructed a benchtop mock-loop circuit consisting of a three-layer flow cell with two enclosed SNM (Supplementary Fig. S2), where the top, middle, and bottom compartments recapitulated the “artery”, “encapsulated islet chamber”, and “vein”, respectively. We subsequently characterized the percentage of cytokines, glucose, and insulin within the different locations of the mock-loop device. Finally, we tested the viability and glucose-insulin response of the SNM-encapsulated mouse islets in the mock-loop circuit with circulating cytokines.

### Substrate preparation

#### Silicon Nanopore Membranes (SNM) architecture and fabrication

Silicon nanopore membranes (SNM) have been prototyped from silicon substrates by MEMS technology as previously reported^23^,^45^,^46^ with some modifications (Fig. 2). Briefly, the process used the growth of a thin SiO_2_ (oxide) layer on 400 μm-thick double side polished (DSP) silicon wafers followed by a low pressure chemical vapor deposition (LPCVD) of polysilicon (~500 nm). The wafers were then specifically patterned, dry oxidized, wet etched, deposited with a second polysilicon layer, and finally blanket etched until 400 nm of polysilicon remained and the underlying vertical oxide layer was exposed. The vertical sacrificial oxide layer defined the critical nanoscale pore size of the membranes. The low temperature oxide (LTO) (~1 μm) was deposited onto polysilicon of the wafers to serve as the hard mask for membrane protection. Deep reactive ion etching (DRIE) removed the backside of each window until membranes were disclosed. Eventually, the sacrificial oxide was etched away in 49% hydrofluoric acid (HF) during the final step of the fabrication process to leave behind open nanoscale slit pores. The wafers were subsequently cut into 1 × 1 cm chips with an effective area of 6 × 6 mm^2^ containing 1500 windows each, with a total of 10^6^ pores per membrane. Each rectangular pore was 7 nm in width, 300 nm in depth, and 2 μm in length. All membranes were cleaned using a conventional “piranha” clean procedure, which involved a 20 min-immersion in 3:1 sulfuric acid (H_2_SO_4_)/hydrogen peroxide (H_2_O_2_) mixture, followed by thorough rinses in deionized (DI) water. Images of SNM were obtained using scanning electron microscope (SEM) (Leo 1550) (Fig. 1).

### Surface modification of SNM with poly(ethylene glycol) (PEG)

SNM were covalently modified with PEG using a previously reported protocol^47^ with some modifications to prevent protein fouling on the membrane surface. The technique used for PEG attachment involved a single reaction step which covalently couples silicon surface silanol group (Si-OH) to a chain of PEG polymer through a trimethoxysilane group forming a Si-O-Si-PEG sequence. Briefly, SNM were immersed in a solution of 3 mM 2-[methoxy(polyethyleneoxy)propyl]trimethoxysilane (PEG-silane) (Gelest: SIM6492.7) in toluene for 2 hr at 70 ^°^C. A series of extensive washing steps involving toluene, ethanol, and DI water were used to rinse away unbounded PEG residue.

### Hydraulic permeability for SNM pore size characterization

An automated mass and pressure measurement system was utilized for characterizing liquid flow through the SNM under a tangential-flow filtration operation^34^. The pore size of the SNM can be related to filtration flow parameters using 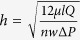 (Eq. 3), where h is pore width, μ is the viscosity, l is the membrane thickness, Q is the volumetric flow rate, n is the number of pores per membrane, w is the pore length, and ΔP is the transmembrane pressure^34^. To assemble the overall system for SNM pore size characterization (Supplementary Fig. S3), air was applied through a syringe pump (Sigma: Z675709) into a water reservoir. Water was circulated by a peristaltic pump (Masterflex: 07551-00) through a differential pressure transducer (Omega: PX429 015GI), a flow cell with enclosed membrane, and returned to the original water reservoir. The flow cell was assembled with the SNM submerged under water to remove air bubbles from all compartments. Specifically, a membrane was positioned with the polysilicon interface facing down with a customized silicone gasket positioned on top of the membrane, followed by the final placement of a filtrate chamber on top of the gasket. All sections were fastened together and secured to the base with hand-tightened hex bolts until gasket was visibly compressed. The ultrafiltrate permeated through the membrane was routed to a liquid collection container that rested on a precision mass balance (Mettler Toledo: XS205). Measurements from the differential pressure transducer and the mass balance were automatically collected with a data acquisition laptop. A typical membrane hydraulic permeability test consisted of 5 ml/min flow rate and 4 pressure cycles (5, 1, 5, and 1 psi) for durations of 150 s each. Using the specifications for pore length, membrane thickness, and total number of pores provided based on individual wafer designs, the average pore size of SNM was calculated using Equation 1. All SNM membranes in this study were surface-modified with PEG and exhibited an average pore size of ~7 nm.

### Assessment of SNM immunoisolation *in vitro*

#### Membrane sieving coefficients under pressure-driven filtration

Fluid was circulated by a peristaltic pump through a circuit that consisted of a differential pressure transducer, a polycarbonate flow cell with enclosed SNM, a three-way valve, and a fluid reservoir (Supplementary Fig.S4). The flow cell consisted of two separate flow cell compartments sandwiching a single SNM and silicone gasket. The top filtrate chamber routed permeated ultrafiltrate to a liquid collection container, whereas the base chamber was connected to a three-way valve. A solution of 3% bovine serum albumin (BSA) (Sigma: A-7030) was used to flush the entire loop prior to the experiment. Solution consisting of mouse cytokines TNF-α (1000 U/ml) (Peprotech: 315-01A), IFN-γ (1000 U/ml) (Peprotech: 315-05), IL-1β (50 U/ml) (Peprotech: 211-11B)^48^, glucose (400 mg/dL) (Sigma-Aldrich: G8270), and insulin (150 mU/L) (Novo Nordisk: 0169-1833-11) in a 3% BSA solution was then switched to the circuit at 5 ml/min with a physiological pressure difference ~2 psi^36^. Ultrafiltrate that permeated through the SNM was collected at various time points for up to 6 hrs and analyzed with the enzyme-linked immunosorbent assays (ELISA) (BD Biosciences: 560478 & 558258; Thermo Pierce: EM2IL1B). The sieving coefficients of solutes across SNM were calculated using 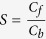 (Eq. 1)^49^, where S is the sieving coefficient, C_f_ is the concentration of the solute in the filtrate, and C_b_ is the molecule concentration in the bulk retentate solution.

### Solute distribution in the mock-loop circuit

We assembled a mock-loop circuit with three flow cell components without cells (Supplementary Fig. S2) to mimic the architecture of the final bioartificial pancreas device. Briefly, two SNM with customized silicone gasket frames were sandwiched in between three flow cell components. The middle flow cell was the encapsulation chamber comprised of a cylindrical chamber separating the two membranes. A peristaltic pump drove the fluid through the top of the flow cell mimicking the “artery”, then over the bottom of the flow cell resembling “vein”, and finally back to the original reservoir. For convective experiments, a three-way valve was used to create flow resistance for a physiological pressure difference ~2 psi between the top and the bottom compartments of the flow cell. Ultrafiltration occurred in the middle encapsulation chamber at this pressure difference. To study the transport of cytokines through the three-layered bioartificial pancreas device, solution consisting of mouse cytokines TNF-α (1000 U/ml), IFN-γ (1000 U/ml), and IL-1β (50 U/ml), glucose (400 mg/dL), insulin (150 mU/L) in 3% BSA was circulated through the circuit at a flow rate of 5 ml/min. Silicon membranes with 1000 nm-wide slit pores (SμM) were used as the control. Solutions were collected and analyzed with ELISA at the end of 6-hr experiments for the top, middle, and bottom chambers.

### Culture of membrane-encapsulated islets in the mock-loop circuit

All procedures described involving isolation of mouse islets were performed in accordance with protocols approved by the Institutional Animal Care and Use Committee (IACUC) at the University of California, San Francisco (UCSF). Mouse islets were isolated from 8 to 10-week-old male B6 mice (Jackson Laboratories) based on previously described protocols^50^. Harvested islets were maintained in suspension culture with RPMI 1640 with L-glutamine and 11.1 mM glucose (Gibco: 11875-093), 10% fetal bovine serum (FBS) (Gibco: 16000), and 1% penicillin-streptomycin (P/S) (UCSF Cell Culture Facility: CCFGK003). A group of 500 mouse islets were introduced into the middle encapsulation chamber of the mock-loop device (Supplementary Fig. S2). To evaluate cell performance with cytokine exposure, the circuit reservoir was replaced with culture medium added with TNF-α (1000 U/ml), IFN-γ (1000 U/ml), and IL-1β (50 U/ml) for 6 hrs. Static culture conditions with or without cytokine exposure were used as the controls. Mouse islets were subsequently isolated for viability testing (2.3.4) and glucose challenge (2.3.5).

### Islet viability

Islet viability was assessed by double staining with fluorescein diacetate (FDA) (Sigma: F7378) and propidium iodide (PI) (Sigma: 287075) as described by protocol (SOP Document: 3104, A02) from National Institute of Allergy and Infectious Diseases (NIAID). Briefly, mouse islets were incubated in phosphate buffered saline (PBS) containing 0.067 μM FDA and 4.0 μM PI for 30 min and extensively washed in PBS to remove excess staining. Images of mouse islets were obtained using laser scanning Nikon Spectral C1si confocal microscope (Nikon Instruments). Viability of islets was calculated based on the ratio between the number of live cells in the islet and the area of that islet.

### Glucose stimulated insulin secretion assay

Mouse islets retrieved from the middle chamber of the mock-loop circuit were rested in RPMI 1640 containing 30 mg/dL glucose (Gibco: 11879) for 15 minutes before exposed to medium containing 300 mg/dL glucose for 15 minutes. After glucose stimulation, the islets were then returned to medium containing 30 mg/dL glucose. Supernatant was collected every 5 minutes during the series of incubations and insulin content was measured with mouse insulin ELISA kits (Mercodia:10-1247-01) and normalized by extracted total protein concentration (Thermo: 78505; 23225).

### Statistical analysis

Sample pairs were analyzed using Student’s t-test. Multiple samples were evaluated with one-way or two-way analysis of variance (ANOVA) followed by Bonferroni and multiple comparison using Graphpad Prism software (San Diego, CA). A p value of <0.05 was accepted as statistically significant for all analyses.

## Additional Information

**How to cite this article**: Song, S. *et al*. Silicon nanopore membrane (SNM) for islet encapsulation and immunoisolation under convective transport. *Sci. Rep.* **6**, 23679; doi: 10.1038/srep23679 (2016).

## Supplementary Material

Supplementary Information
