# Supplementary material for: Silicon nanopore membrane (SNM) for islet encapsulation and immunoisolation under convective transport
Source: Sci Rep. 2016 Mar 24;6:23679. doi: 10.1038/srep23679 (PMC4806308; doi:10.1038/srep23679)

Title:

Silicon nanopore membrane (SNM) for islet encapsulation and immunoisolation under convective transport

Authors:

Shang Song[a], Gaetano Faleo[b,#], Raymond Yeung[a,#], Rishi Kant[a], Andrew Posselt[b], Tejal Desai[a], Qizhi Tang[b], Shuvo Roy[a,*]

[a] Department of Bioengineering and Therapeutic Sciences, University of California - San Francisco, San Francisco, CA, 94158, United States
[b] Department of Surgery, University of California - San Francisco, San Francisco, CA, 94143, United States
[#] These authors contributed equally to this work

* Corresponding author: Department of Bioengineering and Therapeutic Sciences, University of California - San Francisco, Byers Hall, Room 203A, MC 2520, 1700 4th Street, San Francisco, CA 94158, USA.
E-mail address: shuvo.roy@ucsf.edu (S.Roy)
Phone number: 415-514-9666
Fax number: 415-514-9766

Supplementary Information

Extended Data

Figure 1. Conceptual illustration of the implantable intravascular bioartificial pancreas device in the arm of a T1D patient. Transplanted islets will be encapsulated between two SNM sheets mounted on as an arterio-venous (AV) graft. The arterio-venous pressure differential will generate ultrafiltrate that continuously support the islets, which will, in turn, sense glucose levels and produce insulin that will be swpt into the venous blood. The small pore size of the SNM ensures appropriate immunoisolation between the transplanted islets and host.

Figure 2. Schematic diagram of the mock-loop circuit for in vitro assessment of SNM-encapsulated islets under convective conditions. A peristaltic pump circulated liquid through the top compartment of the flow cell, a pressure transducer, a 3-way valve, the bottom compartment of the flow cell, and finally back to the original reservoir. The flow cell was composed of two membranes dividing the flow cell into three compartments, where islets were placed inside the middle chamber. Ultrafiltrate flow occurred within the middle chamber between two semipermeable membranes as the top membrane was adjacent to a high-pressure "arterial" blood channel and the second membrane was adjacent to a low-pressure "vein" blood channel. The 3-way valve was used to create a pressure difference of ~2psi between the top and the bottom compartment mimicking the physiological condition.

Figure 3. Schematic diagram of the hydraulic permeability testing system. Air was applied through a pressure regulator into the liquid reservoir. A peristaltic pump circulated this liquid through the flow cell with enclosed membrane. The flow cell connected to a differential pressure transducer that was automatically controlled by a data acquisition laptop to adjust the transmembrane pressure. The permeated ultrafiltrate was collected into a liquid container on top of a precision mass balance. Data from the differential pressure transducer and the mass balance were automatically collected and stored in a data acquisition laptop.

Figure 4. Schematic diagram of the pressure-driven cytokine filtration testing system. A peristaltic pump circulated liquid through a flow cell that connected to a 3-way valve to establish transmembrane pressure. The permeated ultrafiltrate through the membrane was collected at various time for up to 6 hrs.

Figure 5. Comparison of relative solute size (λ). Experimental relative solute size (mean ± SE) is calculated based on the sieving coefficients for cytokines at 6 hrs. Theoretical values were determined using the Stokes-Einstein's equation [12].

Figure 6. Assessment of solute distribution in the mock-loop system. The mock-loop circuit was composed of two membranes dividing the flow cell into the top, middle, and the bottom compartments. Concentration of solutes from each chamber was assessed at the end of the 6 hr experiment and was expressed as a percentage (mean ± SE) relative to that of the feed solution. Silicon micropore membrane (SμM) consisted of 1000 nm diameter slit pores were used as control. The data showed that the amount of TNF-α, IFN-γ, and IL-1β were significantly reduced to 30%, 35%, and 34% in the middle chamber, whereas small molecules insulin and glucose passed completely (~100%) through SNM under convective flow. However, all molecules including cytokines passed into the middle chamber that were sandwiched between SμM. ($n>3$, *$p < 0.05$).

Extended Data Figures

Figure 1:

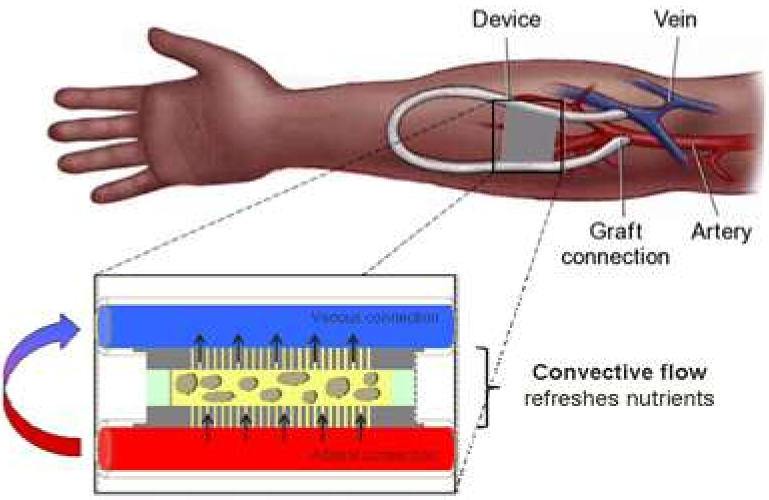

Figure 2:

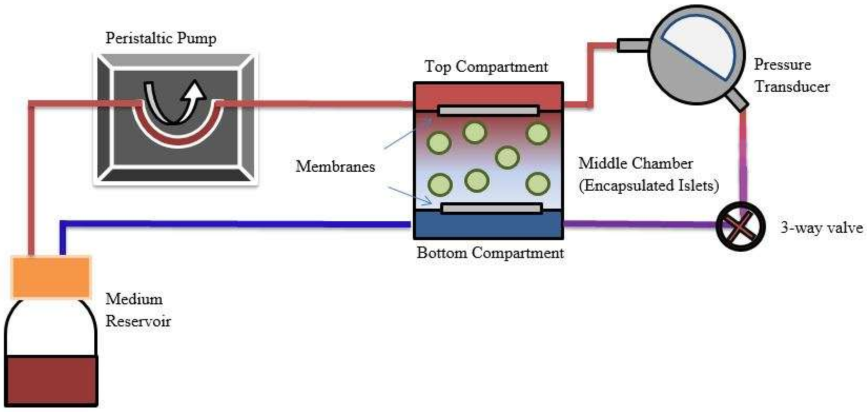

Figure 3:

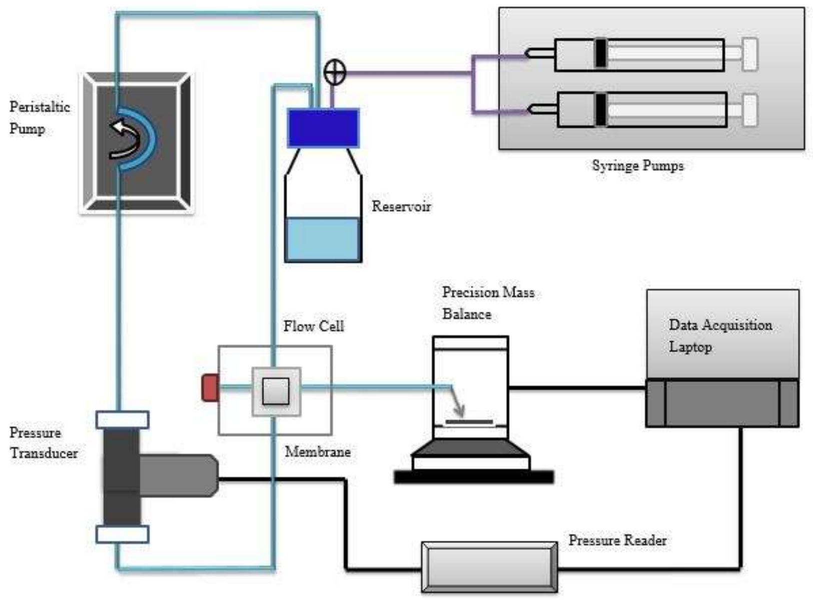

Figure 4:

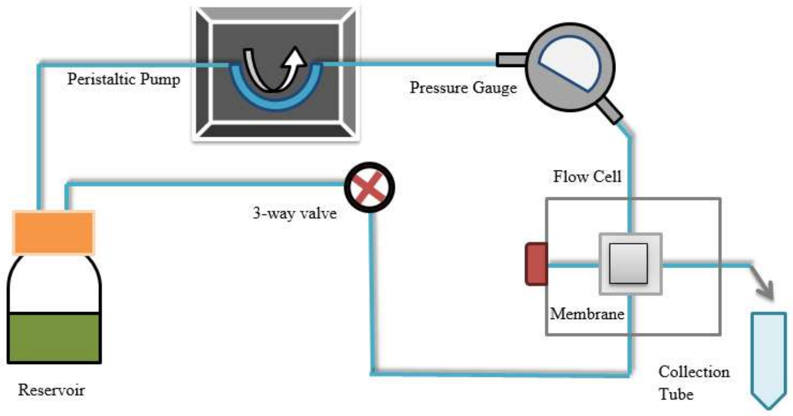

Figure 5:

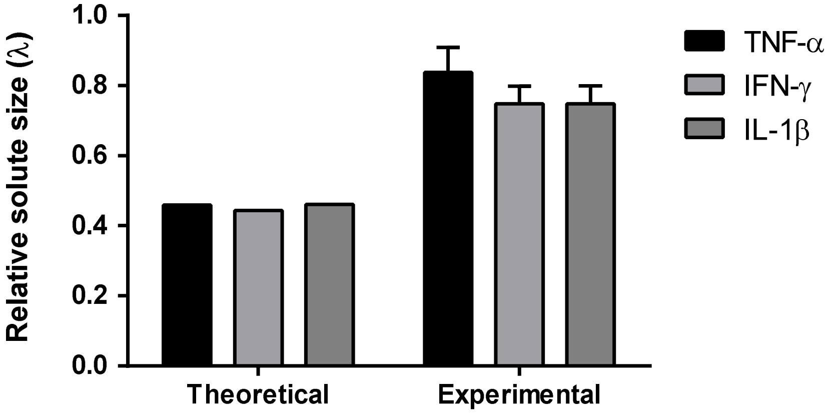

Figure 6:

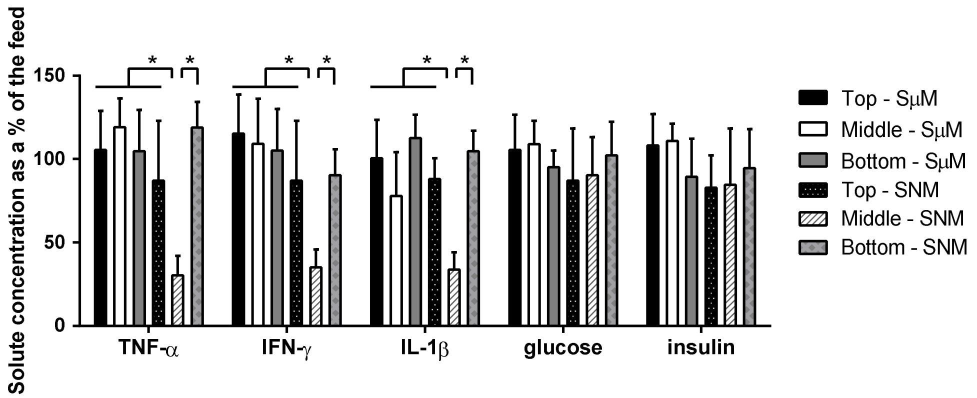

Supplement: Supplementary Information [file srep23679-s1.pdf]
